# Quantitative Magnetic Resonance Imaging and Patient-Reported Outcomes in Patients Undergoing Hip Labral Repair or Reconstruction

**DOI:** 10.3390/jimaging11080261

**Published:** 2025-08-05

**Authors:** Kyle S. J. Jamar, Adam Peszek, Catherine C. Alder, Trevor J. Wait, Caleb J. Wipf, Carson L. Keeter, Stephanie W. Mayer, Charles P. Ho, James W. Genuario

**Affiliations:** 1 Department of Orthopedics, University of Colorado School of Medicine, Aurora, CO 80045, USAcatherine.alder@cuanschutz.edu (C.C.A.); caleb.wipf@cuanschutz.edu (C.J.W.); carson.keeter@cuanschutz.edu (C.L.K.);; 2 UCHealth Steadman Hawkins Clinic, Englewood, CO 80112, USA; 3 Department of Radiology-Diagnostics, University of Colorado School of Medicine, Aurora, CO 80045, USA

**Keywords:** MRI T2 mapping, patient reported outcomes, tissue quality

## Abstract

This study evaluates the relationship between preoperative cartilage quality, measured by T2 mapping, and patient-reported outcomes following labral tear treatment. We retrospectively reviewed patients aged 14–50 who underwent primary hip arthroscopy with either labral repair or reconstruction. Preoperative T2 values of femoral, acetabular, and labral tissue were assessed from MRI by blinded reviewers. International Hip Outcome Tool (iHOT-12) scores were collected preoperatively and up to two years postoperatively. Associations between T2 values and iHOT-12 scores were analyzed using univariate mixed linear models. Twenty-nine patients were included (mean age of 32.5 years, BMI 24 kg/m^2^, 48.3% female, and 22 repairs). Across all patients, higher T2 values were associated with higher iHOT-12 scores at baseline and early postoperative timepoints (three months for cartilage and six months for labrum; *p* < 0.05). Lower T2 values were associated with higher 12- and 24-month iHOT-12 scores across all structures (*p* < 0.001). Similar trends were observed within the repair and reconstruction subgroups, with delayed negative associations correlating with worse tissue quality. T2 mapping showed time-dependent correlations with iHOT-12 scores, indicating that worse cartilage or labral quality predicts poorer long-term outcomes. These findings support the utility of T2 mapping as a preoperative tool for prognosis in hip preservation surgery.

## 1. Introduction

Hip labral tears are a prevalent cause of groin pain and functional limitation in young and active patients, with reported prevalence rates ranging from 22% to 55% in athletes and up to 69% in patients with mechanical hip pain [1,2]. The labrum is a fibrocartilaginous structure that encircles the acetabulum and enhances joint stability and shock absorption while maintaining joint lubrication through a suction seal [3]. Labral pathology significantly impacts joint biomechanics, leading to altered load distribution across the hip joint and contributing to the progression of osteoarthritis if left untreated [4,5]. The economic burden and functional impact on young, active individuals underscores the importance of developing predictive tools for optimal surgical planning.

When conservative treatment measures fail to provide adequate symptom relief, surgical intervention is often indicated as the definitive treatment to relieve pain, restore function, and protect the cartilage from further damage [4,6]. The choice between repair and reconstruction depends largely on the quality and quantity of remaining labral tissue, with repair being preferable when sufficient healthy tissue is available to restore the suction seal [7]. Cartilage quality and labral health are thought to be strong predictors of surgical outcomes, as the integrity of these structures directly influences joint biomechanics and the ability to restore normal hip function [3]. However, accurate assessment of tissue quality preoperatively remains challenging, as the labrum lies within the intra-articular space of the hip capsule, presenting significant obstacles for visualization and assessment outside of the operating room [7].

Magnetic resonance imaging (MRI) with T2 mapping provides quantitative information about the biochemical composition and collagen orientation of cartilage and labral tissue. T2 relaxation time reflects the interaction between water molecules and the collagen matrix, with values serving as biomarkers of tissue integrity [8]. Specifically, T2 mapping measures the transverse relaxation time of hydrogen protons in tissue water, which is influenced by collagen content, organization, and water mobility within the extracellular matrix [9]. In healthy hip cartilage, T2 values typically range from 40 to 50 milliseconds, while damaged cartilage demonstrates elevated values in the 50–60 millisecond range [10,11,12]. Elevated T2 values indicate disrupted collagen architecture or increased water content, as seen in early degeneration, while very low T2 values may indicate calcification or fibrosis [9].

Current literature demonstrates mixed findings regarding the relationship between cartilage quality and patient outcomes following hip preservation surgery. Some studies using dGEMRIC (delayed gadolinium-enhanced MRI of cartilage) have failed to establish clear relationships between quantitative MRI markers and patient-reported outcomes [13,14], while others have found associations between cartilage degradation and functional measures [15,16]. However, T2 mapping offers distinct advantages over contrast-enhanced techniques, providing non-invasive assessment without the need for gadolinium administration [9]. Furthermore, two recent studies found a correlation between T2 mapping values and intraoperative acetabular cartilage quality [17,18]. The gaps in current knowledge include a limited understanding of how preoperative T2 mapping values relate to patient-reported outcomes over time, particularly in the context of different surgical interventions and recovery phases.

Patient-reported outcomes (PROs) are commonly used to assess a patient’s subjective improvement in pain and function pre- and postoperatively, providing crucial insights into treatment effectiveness from the patient’s perspective. Given its wide usage and validation, we elected to use the iHOT-12 [19,20]. The relationship between quantitative tissue quality measures and postoperative PROs remains unclear, particularly for hip labral pathology, where tissue healing patterns may differ from other cartilaginous structures.

To our knowledge, no studies have investigated the association between T2 mapping and PROs before and after hip arthroscopy for labral tears. This gap limits current clinical decision-making, as preoperative predictions of outcomes could significantly improve surgical decision-making and patient counseling. The aim of this study is to assess the relationship between labrum tissue quality as measured by T2 mapping and patient-reported outcomes (PROs) following hip arthroscopy for labral tears. We hypothesize that T2 mapping values will be negatively associated with both baseline and postoperative PROs, such that higher T2 values (indicating poorer tissue quality) will correlate with worse pain and function for up to 2 years postoperatively.

## 2. Materials and Methods

### 2.1. Study Design and Sample

Following IRB approval, patients undergoing hip arthroscopy for labral tear at a single institution with two high-volume hip arthroscopy surgeons between March 2021 and February 2023 were reviewed.

This cohort was reviewed as part of a previous study by the same authors using the following inclusion and exclusion criteria [21]. Inclusion criteria consisted of patients who underwent primary hip arthroscopy surgery with labral repair or reconstruction, who were aged 14–50 years, and who had completed preoperative MRI with T2 mapping following the standard protocol specified below. Patients were excluded for a history of previous ipsilateral hip surgery, traumatic injury (acetabular fracture, hip dislocation, etc.), history of inflammatory arthritis, recent (within four weeks) history of intra-articular injection of a steroid or bioactive agent, and patients who did not have appropriate T2 mapping imaging.

**Figure 1:**
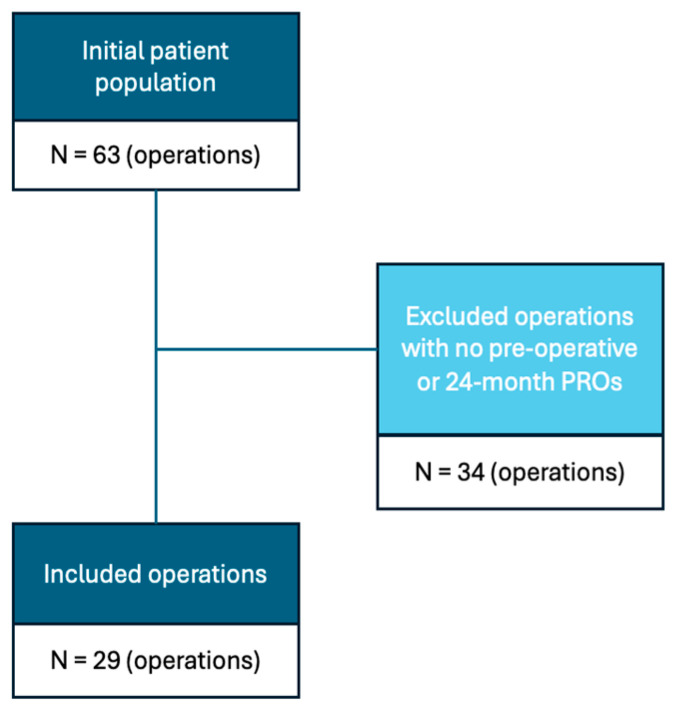
Flowchart of inclusion and exclusion criteria [22,23].

For the present study, an additional exclusion criterion was added for PRO completeness. Any patients who did not have preoperative and 24-month PRO data were excluded. This excluded 34 out of the 63 operations, leaving 29 for analysis (Figure 1).

### 2.2. Preoperative Imaging Technique

Each patient was imaged with a 3.0T (Magnetom Vida, Siemens Medical Solutions, Erlangen, Germany) using an 18-channel Body Flex Coil anteriorly and a 32-channel Spine Coil posteriorly (Siemens Medical Solutions, Erlangen, Germany) prior to hip arthroscopy. The imaging protocol included standard clinical morphologic sequences followed immediately by a sagittal multi-echo spine-echo (MESE) T2-mapping sequence. The T2 mapping sequence (TR/TE 1530.0/13.80–69.00 ms; VS, 0.5 × 0.5 × 3.0 mm^3^; Slices, 20; Slice thickness 3.0 mm; FOV, 160 mm; AT, 4:51 min; FOV read 150 mm; Flip Angle 180 degrees) was acquired in the sagittal plane for optimal mapping of the anterior lateral articular cartilage, the area of most common pathology for labral and cartilage lesions in femoroacetabular impingement. The T2 mapping sequence was acquired after the standard MRI images were obtained and the patient had been recumbent for a prolonged period. The T2 mapping images were derived from the Siemens MapIT software algorithm (Seimens Healthineers, Erlangen, Germany).

### 2.3. Data Collection

**Figure 2:**
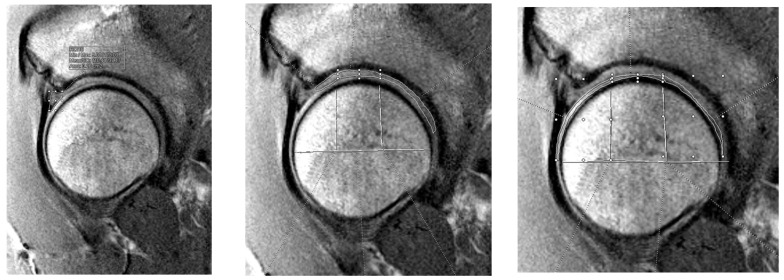
T2 mapping segment identification for labrum (**left**), acetabular cartilage (**center**), and femoral cartilage (**right**). The average T2 value was taken for the area identified as labral tissue. The acetabular and femoral cartilage were divided into anterior, superior, and posterior zones.

Three individual reviewers, one fellow and two medical students under the supervision of a board-certified musculoskeletal radiologist, were blinded to patients’ surgical information and independently analyzed each patient’s preoperative MRI. Each reviewer performed three separate sequencing analyses with the Syngo.via T2 mapping software (Siemens Healthineers, Erlangen, Germany) on the sagittal cut analysis of each patient’s preoperative MRI. All MRIs were obtained preoperatively within three months of the surgery, except for three patients whose MRIs were completed more than three months prior to surgery (four months, eight months, and eighteen months). The sagittal plane was selected to optimize the visualization of the labrum, articular cartilage, and chondral-labral interface in the anterior lateral/superior quadrant, as these tissues are orthogonal to, and consequently most reliably seen, in short-diameter cross-section on sagittal images. Opting for the sagittal plane provided the best resolution, least partial volume averaging, and least magic angle artifact from the sphericity of the tissues of interest. Each reviewer independently selected two to three sagittal cuts from each patient’s MRI for analysis. The selection criteria for these cuts were as follows: (1) the highest definition of labrum morphology and (2) clear partitions between femoral head cartilage and acetabular cartilage. All reviewers analyzed the same anatomical regions but independently selected the specific cuts that best met these criteria for each patient. Structures that were sequenced included the hip labrum (Figure 2 left), acetabular cartilage divided into three separate zones (anterior, superior, and posterior) (Figure 2 center), and the femoral head cartilage divided into three separate zones (anterior, superior, and posterior) (Figure 2 right). Each zone was manually divided and corresponded to zones 2, 3, and 4 in the acetabular and femoral head zones, as described by Iliziliturri et al. [24]. The average T2 mapping value for the labrum and each respective zone on the acetabulum and femoral head was recorded.

Intraclass correlation values were calculated for the mapping values. There was excellent agreement between the raters at the labrum (ICC2k = 0.905, 95% CI [0.724, 0.958], *p* < 0.001), acetabular cartilage (ICC2k = 0.936, 95% CI [0.769, 0.974], *p* < 0.001), and femoral cartilage (ICC2k = 0.936, 95% CI [0.769, 0.974], *p* < 0.001).

Baseline and postoperative international hip outcome tool (iHOT-12) scores were retrospectively collected from the patient’s medical record at baseline, and 3, 6, 12, and 24 months postoperatively. Additional demographic data collected included age, sex, body mass index (BMI), laterality of surgery, surgical procedure performed (repair vs. reconstruction), and concurrent procedures. Patients were grouped based on the type of labral procedure performed, as determined by intraoperative findings and surgeon decision-making.

### 2.4. Statistical Analyses

Multivariate mixed linear models (MLMs) were used with a three-way interaction effect of mapping value, location, and time. These models were used to assess the relationship between T2 mapping values and iHOT-12 scores, with the patient included as a random effect. Analyses were stratified by surgical procedure (labral repair vs. labral reconstruction). Within each group, separate models were constructed to evaluate the effect of T2 values from the labrum, acetabular cartilage, and femoral cartilage on iHOT-12 scores at the following timepoints: preoperative, and 3, 6, 12, and 24 months postoperative. Each model estimated a regression coefficient (β) representing the linear association between the imaging parameter and iHOT-12 score at each timepoint. For every regression coefficient estimate, the corresponding 95% confidence interval (CI) was reported. Statistical significance was determined at α = 0.05.

## 3. Results

Twenty-nine operations were included in the analysis: twenty-two labral repairs and seven labral reconstructions. No significant differences in group demographics were observed (Table 1). The only missing iHOT-12 scores were four 3-month postoperative data points.

### 3.1. T2 Mapping Results—All Patients

**Figure 3:**
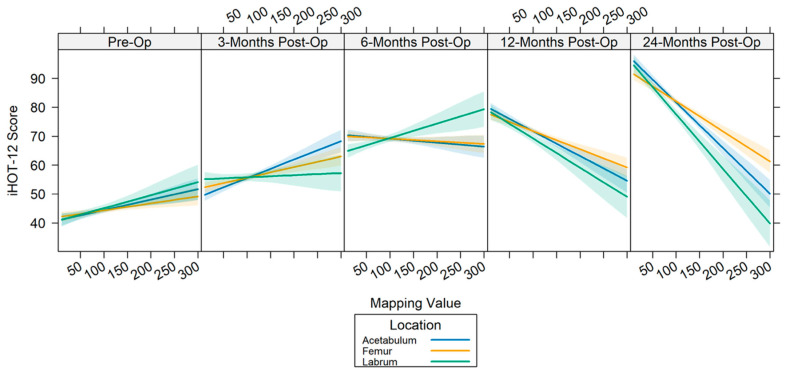
Line plots depicting the relationship between cartilage quality and iHOT-12 scores at each timepoint by tissue type.

The T2 mapping values for cartilage and labral tissue were positively associated with the baseline iHOT-12 scores (acetabular β = 0.036, *p* = 0.001; femoral β = 0.024, *p* = 0.011; and labral β = 0.045, *p* = 0.006) (Table 2). At the mid-term follow-up, cartilage T2 values were positively associated with the 3-month iHOT-12 scores (acetabular β = 0.064, *p* < 0.001; femoral β = 0.037, *p* < 0.001) but not at 6 months (*p* > 0.05). Conversely, labral T2 values were only significantly associated with iHOT-12 scores at 6 months (labrum β = 0.05, *p* = 0.002). The T2 mapping values of cartilage and labral tissue were negatively associated with the 12-month (acetabular β = −0.086, *p* < 0.001; femoral β = −0.063, *p* < 0.001; labral β = −0.101, *p* < 0.001) and 24-month (acetabular β = −0.158, *p* < 0.001; femoral β = −0.104, *p* < 0.001; labral β = −0.189, *p* < 0.001) postoperative iHOT-12 scores (Table 2, Figure 3).

### 3.2. T2 Mapping Results—Labral Repair Group

For the labral repair group, no significant associations were observed between T2 values at any location and baseline iHOT-12 scores (*p* > 0.05 for all). Positive associations were observed for cartilage T2 values with the 3-month iHOT-12 scores (acetabular β = 0.115, *p* < 0.001; femoral β = 0.062, *p* < 0.001) but not the 6-month scores (Table 3). Labral T2 values demonstrated positive associations with the 6-month iHOT-12 scores (labral β = 0.087, *p* < 0.001), but not the 3-month scores (Table 3). Significant negative associations were observed between T2 mapping values at all locations and the iHOT-12 scores at 12 months (acetabular β = −0.139, *p* < 0.001; femoral β = −0.095, *p* < 0.001; labral β = −0.132, *p* < 0.001) and 24 months (acetabular β = −0.136, *p* < 0.001; femoral β = −0.084, *p* < 0.001; labral β = −0.160, *p* < 0.001) (Table 3).

### 3.3. T2 Mapping Results—Labral Reconstruction Group

In the reconstruction group, labral T2 values were significantly associated with the preoperative iHOT-12 scores (labral β = 0.139, *p* < 0.001) but not at the cartilage mapping locations (*p* > 0.05) (Table 4). T2 mapping values at all cartilage locations were positively associated with the 6-month iHOT-12 scores (acetabular β = 0.105, *p* < 0.001; femoral β = 0.075, *p* = 0.001; labral β = 0.098, *p* = 0.012), and negatively associated with the 24-month postoperative iHOT-12 scores (acetabular β = −0.449, *p* < 0.001; femoral β = −0.388, *p* < 0.001; labral β = −0.510, *p* < 0.001) (Table 4). No significant associations were observed between T2 values at any location and the iHOT-12 scores at 3 months or 12 months postoperatively (*p* > 0.05 for all).

## 4. Discussion

In the present study, preoperative iHOT-12 scores were positively associated with higher T2 mapping values across all cartilage locations, suggesting that patients with poorer cartilage quality (higher T2 values) reported better baseline function and pain scores. Conversely, at the 12- and 24-month follow-up, a negative association was observed, indicating that patients with worse cartilage quality (higher T2 values) demonstrated poorer long-term outcomes following surgery. These findings persisted when patients were stratified by labral treatment type, suggesting that T2 values may be a reliable predictor for patients undergoing labral repair or reconstruction.

This study demonstrates a significant association between worse cartilage quality and poorer patient-reported outcomes following hip labral repair or reconstructions. The findings suggest that better cartilage quality (lower T2 values) correlates with superior 12- and 24-month postoperative iHOT-12 scores in both surgical groups. However, the paradoxical baseline finding requires careful interpretation and contrasts with previous reports that have examined the relationship between quantitative MRI markers of cartilage health and patient outcomes.

Previous studies using delayed gadolinium-enhanced MRI of cartilage (dGEMRIC) have yielded mixed results regarding the relationship between cartilage quality and outcomes following hip preservation surgery [15,16,19]. These conflicting findings may be attributed to differences in measurement techniques (contrast-enhanced vs. non-contrast T2 mapping), patient populations (primary vs. revision surgery, varying pathology severity), surgical approaches (arthroscopic vs. open procedures), and follow-up timeframes. The present study’s use of T2 mapping offers advantages over contrast-enhanced techniques, providing quantitative assessment without gadolinium administration and potentially greater sensitivity to early cartilage changes.

The present study demonstrates that postoperative recovery time and labral treatment type influence the relationship between cartilage quality and iHOT-12 scores. Labral repair patients demonstrated robust associations between T2 mapping values and outcomes across all three cartilage regions at 12 and 24 months, with less consistent associations at earlier timepoints. This temporal pattern may be attributable to the natural progression of cartilage recovery and remodeling following surgical correction of labral tears. Early postoperative outcomes may be more influenced by acute inflammatory responses, soft tissue healing, and rehabilitation progress, whereas the 12- and 24-month timepoints capture a more stable state of tissue adaptation and functional recovery.

Conversely, in the reconstruction group, significant negative associations between T2 mapping values and iHOT-12 scores did not appear until the 24-month postoperative timepoint. This delayed pattern may reflect the biological and mechanical differences inherent in reconstructed labral tissue compared to repaired native tissue. Labral reconstruction involves the use of allograft or autograft material, which requires longer integration periods and may have different biomechanical properties compared to native labral tissue [4]. Additionally, patients requiring reconstruction may present with more advanced chondral damage and more complex pathology, potentially influencing the timeline of functional recovery.

The counterintuitive baseline findings, where higher T2 values (worse cartilage quality) were associated with better preoperative iHOT-12 scores, warrant careful consideration. Several mechanisms may explain this phenomenon. First, patients with more advanced cartilage degeneration may have adapted their activity levels and pain expectations over time, leading to relatively higher self-reported function scores despite objective tissue damage. Second, concurrent pathology such as labral hypertrophy or synovial inflammation may contribute to symptoms independent of cartilage T2 values. This finding underscores the multifactorial nature of hip pain and the importance of comprehensive assessment beyond structural imaging alone.

The data suggest that baseline cartilage quality influences long-term iHOT-12 scores following hip labral surgery, although it represents one of multiple factors affecting outcomes. While there was a clear association between lower T2 scores (indicating better tissue quality) and higher 24-month iHOT-12 scores across both surgical groups, other patient-specific, clinical, and surgical factors not controlled for in this study likely contribute significantly to outcomes. These may include baseline activity levels, expectations, psychological factors, rehabilitation compliance, concurrent pathology, and surgical technique variations. Therefore, while baseline cartilage quality appears to be an important predictive factor, it should not be considered the primary determinant of surgical outcomes.

This study has several limitations worth discussing. First, the follow-up duration of 24 months may not fully capture the complete trajectory of cartilage healing and remodeling, as these processes may continue beyond the study timeframe. Second, the sample size is relatively modest, particularly in the reconstruction group (n = 7), limiting statistical power and the generalizability of findings. As a pilot study, these findings establish preliminary evidence for T2 mapping as a prognostic tool in hip preservation surgery, but require validation in larger, multi-site cohorts. Third, the limitations of the labral measurement technique must be considered. Variable image quality and occasional extensive labral damage made labral identification and segmentation challenging on T2 mapping sequences. While this limitation was addressed through multiple independent reviewers and regression modeling that accounts for measurement variability, the potential for inaccurate T2 mapping data remains and may impact the study results. Fourth, this study did not control for potential confounding variables (rehabilitation protocol/compliance, physiological state, etc.). While previous work has begun to establish the relationship between bony anatomy and labral pathology, we did not control for variables such as rehabilitation protocols or baseline activity levels, both of which may influence outcomes independent of cartilage quality [21]. Lastly, we did not specifically assess epiphyseal closure status in our younger patients, which could potentially influence cartilage T2 values. However, the mixed effects modeling approach accounts for individual patient differences, and the clinical relevance of including older adolescents reflects real-world practice patterns. Future studies should incorporate a comprehensive assessment of acetabular and femoral morphology alongside T2 mapping data.

## 5. Conclusions

Despite these limitations, the results provide valuable insights for clinical practice. The finding that cartilage quality influences long-term outcomes, particularly at 12 and 24 months postoperatively, suggests that preoperative T2 mapping may have utility in patient counseling and outcome prediction, although it should be interpreted within the broader clinical context rather than as a standalone predictive tool given the inconsistent relationships at earlier timepoints and paradoxical baseline findings. Future research should focus on developing comprehensive predictive models that incorporate T2 mapping data alongside other relevant factors such as baseline demographics, activity levels, and psychosocial variables, while longer-term follow-up studies are needed to determine whether the relationships observed at 24 months persist over time and to assess the predictive utility of T2 mapping for more distant outcomes such as progression to total hip arthroplasty.

In summary, preoperative cartilage quality as measured by T2 mapping demonstrates significant associations with patient-reported outcomes following hip labral repair and reconstruction, though the relationship varies by surgical procedure type and postoperative timeframe. While these findings suggest potential clinical utility for outcome prediction, the complex and sometimes counterintuitive relationships observed underscore the need for continued research to fully understand the role of quantitative imaging in hip preservation surgery.

## Figures and Tables

**Table 1:** Demographic data.

Characteristic	Overall (N = 29)	Repair (N = 22)	Recon (N = 7)	*p*-Value
**Age (yrs)**				0.183
Mean ± SD	32.5 ± 10.8	30.8 ± 11.2	37.6 ± 8.0	
Range	14–50	14–50	23–45	
**Sex, n (%)**				0.755
Male	15 (51.7)	11 (50)	4 (57.1)	
Female	14 (48.3)	11 (50)	3 (42.9)	
**BMI (kg/m^2^)**				0.196
Mean ± SD	24.3 ± 2.6	23.9 ± 2.5	25.5 ± 2.6	
Range	19.2–29.3	19.2–27.5	21.5–29.3	
**Laterality, n (%)**				0.108
Left	16 (55.2)	14 (63.6)	2 (28.6)	
Right	13 (44.8)	8 (36.4)	5 (71.4)	

**Table 2:** Association between T2 mapping values and iHOT-12 scores in all patients.

		Baseline	3 Months	6 Months	12 Months	24 Months
**Labrum**	**β**	0.045	0.007	0.050	−0.101	−0.189
	95% CI	(0.009, 0.081)	(−0.03, 0.045)	(0.014, 0.086)	(−0.143, −0.058)	(−0.236, −0.142)
	*p*-value	0.006	>0.90	0.002	<0.001	<0.001
**Acetabulum**						
	**β**	0.036	0.064	−0.014	−0.086	−0.158
	95% CI	(0.011, 0.061)	(0.038, 0.099)	(−0.039, 0.011)	(−0.112, −0.059)	(−0.189, −0.127)
	*p*-value	0.001	<0.001	0.591	<0.001	<0.001
**Femur**						
	**β**	0.024	0.037	−0.009	−0.063	−0.104
	95% CI	(0.004, 0.044)	(0.016, 0.058)	(−0.03, 0.011)	(−0.085, −0.041)	(−0.129, −0.079)
	*p*-value	0.011	<0.001	0.763	<0.001	<0.001

CI = Confidence Interval. Note: Each timepoint and measurement location is a separate univariate mixed linear model.

**Table 3:** Association between T2 mapping values and iHOT-12 scores in patients with labral repair.

		Baseline	3 Months	6 Months	12 Months	24 Months
**Labrum**	**β**	−0.010	0.030	0.087	−0.132	−0.160
	95% CI	(−0.051, 0.031)	(−0.014, 0.073)	(0.046, 0.129)	(−0.183, −0.081)	(−0.216, −0.104)
	*p*-value	0.9	0.336	<0.001	<0.001	<0.001
**Acetabulum**						
	**β**	−0.012	0.115	0.002	−0.139	−0.136
	95% CI	(−0.042, 0.017)	(0.084, 0.146)	(−0.028, 0.031)	(−0.171, −0.107)	(−0.173, −0.099)
	*p*-value	0.807	<0.001	>0.90	<0.001	<0.001
Femur						
	**β**	−0.011	0.062	0.009	−0.095	−0.084
	95% CI	(−0.034, 0.012)	(0.038, 0.086)	(−0.014, 0.032)	(−0.121, −0.070)	(−0.113, −0.055)
	*p*-value	0.676	<0.001	0.837	<0.001	<0.001

CI = Confidence Interval. Note: Each timepoint and measurement location is a separate univariate mixed linear model.

**Table 4:** Association between T2 mapping values and iHOT-12 scores in patients with labral reconstruction.

		Baseline	3 Months	6 Months	12 Months	24 Months
**Labrum**	**β**	0.139	−0.033	0.098	−0.083	−0.51
	95% CI	(0.056, 0.221)	(−0.121, 0.055)	(0.015, 0.181)	(−0.168, 0.001)	(−0.61, −0.41)
	*p*-value	<0.001	0.867	0.012	0.056	<0.001
**Acetabulum**						
	**β**	0.049	−0.026	0.105	0	−0.449
	95% CI	(−0.005, 0.103)	(−0.081, 0.028)	(0.051, 0.159)	(−0.056, 0.057)	(−0.513, −0.385)
	*p*-value	0.099	0.691	<0.001	>0.90	<0.001
**Femur**						
	**β**	0.04	−0.014	0.076	−0.003	−0.388
	95% CI	(−0.011, 0.091)	(−0.065, 0.037)	(0.026, 0.127)	(−0.056, 0.05)	(−0.447, −0.329)
	*p*-value	0.208	>0.90	0.001	>0.90	<0.001

CI = Confidence Interval. Note: Each timepoint and measurement location is a separate univariate mixed linear model.

## Data Availability

The data underlying this article will be shared on reasonable request to the corresponding author.

## Abbreviations

The following abbreviations are used in this manuscript:

MRI	Magnetic Resonance Imaging
iHOT-12	International hip outcome tool—12 question version
dGEMRIC	Delayed gadolinium-enhanced MRI of Cartilage
PRO	Patient reported outcome
MESE	Multi-echo spin-echo
TR	Repetition time
TE	Echo time
VS	Voxel size
FOV	Field of view
AT	Acquisition time
MLM	Mixed linear models
CI	Confidence interval
